# Activity of Vip3Aa1 against *Periplaneta Americana*

**DOI:** 10.1515/biol-2020-0014

**Published:** 2020-03-24

**Authors:** Wenbin Liu, Lirong Wu, Jie Wang, Xiaobo Li, Xiaobao Jin, Jiayong Zhu

**Affiliations:** 1 School of Pharmaceutical Sciences, Southern Medical University,1023 Shatai South Road, Guangzhou 510515, P. R. China; 2 Guangdong Provincial Key Laboratory of Pharmaceutical Bioactive Substances, Guangdong Pharmaceutical University, Guangzhou 510006, P. R. China

**Keywords:** *Bacillus thuringiensis*, vegetative insecticidal proteins, *Periplaneta americana*, *Blattella germanica*, protease

## Abstract

*Bacillus thuringiensis* (*Bt*) is a well-known entomopathogen. In this study, we cloned the *vip3Aa1* gene from *Bt* strain GIM1.147 and investigated the insecticidal activity of *Bt* Vip3Aa1 protein produced by *Escherichia coli* against *Periplaneta americana* and *Blattella germanica*. The results showed that purified Vip3Aa1 exhibited an LC_50_ at 24 h against *P. americana* and *B. germanica* of 0.182 mg·ml^-1^ and 0.276 mg·ml^-1^, respectively. Investigations of its mode of action showed that Vip3Aa1 could be proteolyzed into a 62-kDa toxic protein by *P. americana* gut-soluble proteases. In addition, Vip3Aa1 caused severe damage to the columnar colon and the midgut, as observed through hematoxylin-eosin staining and scanning electron microscopy. The 62-kDa activated Vip3Aa1 protein could form ion channels in the colon and the midgut in vitro. Based on protease activity analysis, Vip3Aa1 at concentrations of 0.125 mg·ml^-1^ and 0.031 mg·ml^-1^ could downregulate the activities of glutathione S-transferase, α-NA esterase, trypsin, and chymotrypsin. This report provides the first description of the activity of Vip3Aa1 toxins toward *P. americana* and *B. germanica* and demonstrates that the mechanism through which Vip3Aa1 kills *P. americana* and *B. germanica* differs from that involved in the killing of lepidopteran insects.

## 1 Introduction

The cockroach species *Periplaneta americana* and *Blattella germanica* exhibit very strong breeding ability and environmental adaptability, and as a result are found as pests in households, hospitals and residential areas worldwide. As they carry viral and bacterial pathogens on their bodies and in their feces, these pests can cause serious health problems, including poisoning, diarrhea, dysentery, asthma and allergies [1].

The alimentary tract of *P. americana* is mainly divided into three regions, the foregut, midgut and colon. The foregut consists of the esophagus, crop and proventriculus. The crop is covered by a layer of well-defined squamous epithelium, the epidermis has thorns on its surface, and the squamous epithelial cells fold to form a crest. The midgut and the columnar colon have a similar morphology, but the ridge is thicker in the columnar colon than in the midgut. A peritrophic membrane covers the surface of the epithelial cells and protects these cells from food abrasion and pathogenic microorganisms. The epithelium is composed of columnar cells, goblet cells and brush border membranes, and the columnar cells are covered with numerous microvilli.

At present, the primary method used for controlling cockroaches involves the use of chemical pesticides (organophosphates, pyrethroids, and carbamates), which can accumulate in organs and fat, causing chronic poisoning and damage in animal species including humans. In addition, the development of insecticide resistance in cockroaches is a serious problem related to the control of these insects [2]. Therefore, the development of a new and more sustainable approach for the reduction or management of cockroach populations is important, and the use of biological pesticides and their metabolites instead of chemical pesticides for cockroach control is a potentially promising approach because these could reduce risks to human health and provide a longer-term effect.

*Bacillus thuringiensis* (*Bt*), a gram-positive bacillus bacteria found throughout the world, can produce toxins that are harmful to a variety of insects and other invertebrates. During its growth, *Bt* can synthesize many types of active compounds, including vegetative insecticidal proteins (Vips), secreted insecticidal proteins [3], chitinase, and s-layer proteins [4]. Vip3 protein, which is currently the most studied Vip protein, shows high insecticidal activity against lepidopteran insects, such as *Helicoverpa armigera* [5], *Spodoptera exigua* [6], *Spodoptera frugiperda* [7], *Heliothis virescens* [8], *Plutella xylostella*, *Spodoptera litura* [9], *Agrotis ipsilon* [10] and other agricultural pests. Although the mode of action of Vip3 remains to some extent controversial, it is widely accepted that Vip3 protein exerts its toxic action through a number of processes, including: activation by midgut protease, binding to some receptor, and pore formation [11]. In recent studies, some researchers report that Vip3 exerts toxicity by inducing apoptosis.

In this study, we sequenced and cloned the *vip3Aa1* gene and produced Vip3Aa1 protein. We also performed the first evaluation of the activity of the Vip3Aa1 toxin against *P. americana* and *B. germanica* and investigated the activation process, histopathological effects and pore formation of Vip3Aa1 in the midgut and columnar colon of *P. americana*.

## 2 Materials and methods

### 2.1 Strains and growth conditions

The *Bt* strain (GIM 1.147) used in this study was obtained from the Guangdong Culture Collection Center and maintained in nutrient agar medium (1.0% peptone, 0.3% beef extract, 0.5% NaCl, and 1.5% agar, pH 7.4). *E. coli* DH-5α and *E. coli* BL21(DE3) were purchased from Takara (Japan) and maintained in LB medium (1% peptone, 0.5% yeast extract, 1.0% NaCl, and 1.5% agar, pH 7.4). *P. americana* and *B. germanica* were purchased from the Guangdong Provincial Centers for Disease Control and Prevention. The insects were maintained in the rearing facility at 28±1°C with 65±5% relative humidity and a photoperiod of 12:12 light/dark and given mouse food.

### 2.2 Expression of vip3Aa1 in E. coli

The *Bt* strain GIM 1.147 was maintained in nutrient agar medium, and DNA was isolated using a TIANamp Bacteria DNA Kit (Tiangen Biotech Co., Ltd., China). The *vip3Aa1* gene (GenBank accession number: JQ228435.1) was amplified using a pair of primers, which were designed based on the *vip3Aa1* gene with the addition of a 6× His-tag sequence and thus had the following sequences: F-CGC**GGATCC**ATGAACAAGAATAATACTAA and R-CCGCTCGAGTTAATGGTGATGGTGATGATGCTTAATAGA-GACATCGT. A *BamH I* (bold) restriction enzyme site was introduced upstream of the *vip3Aa1* gene, and an *Xho I* (italic) restriction enzyme site and hexahistidine tag (underlined) were introduced downstream of the *vip3Aa1* gene. A 2370-bp fragment was amplified and cloned into a T-vector (pMD-20, Takara, Japan) and then sequenced by Invitrogen Corporation. The *vip3Aa1* gene was excised from pMD-20 using the *Bam* I and *Xho* I restriction enzymes and then cloned into the *E. coli* expression vector pET21b. The recombinant plasmid pET21b-vip3Aa1 was transformed into *E. coli* BL21(DE3), and pET21b-Vip3Aa1/BL21 *E. coli* grown overnight, diluted 100 fold with LB medium and grown to an optical density of 0.6 (at 600 nm, OD 600) at 37℃.

Expression of Vip3Aa1 protein was induced with 0.1 mmol·l^-1^ isopropyl-β-D-thiogalactoside (IPTG) for 24 h at 20°C. After fermentation, the bacterial cells were recovered by centrifugation (8000 g, 10 min, 4°C) and resuspended in 40 ml lysis buffer (25 mmol·l^-1^ Tris-HCl, pH 8.0, 300 mmol·l^-1^ NaCl, and 5 mmol·l^-1^ β-mercaptoethanol). After incubation for 30 min at 37°C, the lysate was subjected to ultrasonic fragmentation for 12 min at 4°C (250 W, work 6 s, off 6 s). The sonicated extracts were centrifuged at 12,000 g and 4°C for 30 min, and the supernatant was applied to a 18 mL HisTrap FF affinity column (GE Healthcare). The column was washed with 100 ml binding buffer (20 mM Tris-HCl, 400 mM NaCl, 50 mM imidazole; pH 7.4) to remove any nonspecific binding, and Vip3Aa1 protein was separated with a linear imidazole gradient from 50 mM to 1 M imidazole for 15min. The fractions were collected in tubes, desalted and freeze-dried.

### 2.3 Insect toxicity bioassays

Ten adult *P. americana* (13 instar) specimens and 15 adult (7 instar) *B. germanica* specimens were used in the bioassay. The purified toxins were diluted in phosphate-buffered saline (PBS) at pH 7.4, and PBS alone was used as a control. *P. americana* or *B. germanica* were fasted for 24 h, placed in a Petri dish with 1 ml of toxin solution (2 mg·ml^-1^, 0.5 mg·ml^-1^, 0.125 mg·ml^-1^, or 0.03125 mg·ml^-1^) and incubated under the above-mentioned rearing conditions. The mortality of the insects was scored after 12 h, 24 h, and 36 h, and the LC_50_ was calculated using the Probit method [12]. The experiment was repeated three times. The full *P. americana* alimentary tract (include foregut, midgut, colon) were carefully excised, and the length objectively measured by a ruler.

### 2.4 Gut juice preparation, proteolysis of Vip3Aa1 and sodium dodecyl sulfate-polyacrylamide gel electrophoresis (SDS-PAGE)

Proteinases were obtained from the midguts of *P. americana*. The *P. americana* specimens were cold immobilized and dissected, and their midgut contents were extracted using a pipette and centrifuged at 4°C and 12,000 g for 20 min. The supernatant was collected and used for proteolytic analysis. The protein concentrations were determined using the Bradford assay. Purified Vip3Aa1 (1 mg·ml^-1^) was dissolved in PBS and incubated with gut juice (0.5 mg·ml^-1^), trypsin (0.250%) or chymotrypsin (0.250%) for different time periods. The protease activity was then terminated by the addition of 0.1 mmol·l^-1^ PMSF, and the reaction products were separated by 10% SDS-PAGE.

### 2.5 Preparation and sectioning of insect tissues

As described in the section 2.3, *P. americana* specimens were fed purified PBS or 0.125 mg·ml^-1^ Vip3Aa1 protein solution for 24 h. The *P. americana* guts (crop, midgut, and columnar colon) were then excised, preserved in 10% formol, dehydrated using solutions with different ethanol concentrations (50%, 70%, 85%, 95% and 100%), vitrified by dimethylbenzene, and fixed in 52-54°C paraffin wax. The embedded gut sections were sectioned longitudinally at a thickness of 4 μm. After deparaffinization and hydration, the sections were stained with hematoxylineosin (HE) and prepared for photomicroscopy.

### 2.6 Scanning electron microscopy (SEM)

Guts (crop, midgut, and columnar colon) were collected in cold 2.5% glutaraldehyde and postfixed with 1% osmium acid. The tissues were then dehydrated using solutions with different concentrations of ethanol (30%, 50%, 70%, 80%, 90% and 100%) and subjected to CO_2_ critical point fixation. After tissue surface was sputter-coated with gold, the tissues were viewed using a JEM1400 transmission electron microscope at 120 kV (JEOL, Japan).

### 2.7 Immunolocalization analysis

For the pore formation activity and immunolocalization analyses, full-length 88-kDa Vip3Aa1 was hydrolyzed by trypsin to generate 62-kDa activated Vip3Aa1 (J-Vip3Aa1), and J-Vip3Aa1 was then further purified using a Superdex 75 column.

FITC was dissolved in DMSO at a concentration of 1 mg·ml^-1^, and J-Vip3Aa1 was dissolved in Na_2_CO_3_-NaHCO_3_ buffer to a concentration of 2 mg·ml^-1^ [13]. A mixture of the FITC and J-Vip3Aa1 solutions was then prepared, and the pH was adjusted to 8.5 using sodium hydroxide solution. The solution was stirred overnight at 4°C and loaded onto a Sephadex G-25 column to remove free FITC. The peaks were collected, and the fluorescence detected using a fluorescence imaging system (Tiangen Biotech Co., China). The columnar colon, midgut, and crop tissues of healthy *P. americana* were separately sectioned at 10 μm under a Leica freezing microtome (Leica, Germany). The sections were then fixed in cold acetone for 10 min, subjected to three 5-min washes with 0.02 M PBS and blocked with 2% BSA for 60 min. The sections were subsequently incubated with FITC-J-Vip3Aa1 at 37°C for 30 min and subjected to three 5-min washes with 0.02 M PBS. The nuclei were counterstained with diamidino-2-phenylindole (DAPI), and the sections were sealed with nail polish and observed under a fluorescence microscope (Leica DMI3000B, Germany).

### 2.8 Pore formation activity of Vip3Aa1

*P. americana* were excised, and the columnar colons, midguts, and crops were removed. Ten grams of tissue was added to 33 ml of buffer A (300 mM mannitol and 10 mM Tris-HCl, pH 7.1). The samples were homogenized, CaCl_2_ was added to a final concentration of 10 mM, and the samples were incubated for 15 min at 4°C. The homogenate was centrifuged at 3000 g and 4°C for 15 min, and the supernatant was collected and centrifuged at 28,000 g and 4°C for 15 min. The precipitate was resuspended in 4 ml of buffer B (50 mM mannitol and 10 mM Tris-HCl, pH 7.1) and centrifuged at 28,000 g and 4°C for 30 min. The resulting brush border membrane vesicle (BBMV) pellet was resuspended in buffer A to obtain 1.0 mg·ml^-1^ BBMVs.

The channel activity was assayed based on the fluorescence quenching of the voltage-sensitive cyanine dye 3,3’-dipropylthiodicarbocyanine iodide DiSC3(5). BBMVs were incubated with buffer, 100 μM valinomycin or 4 μg ml^-1^ J-Vip3Aa1 at 4°C for 30 min, and 6 μl of 1 mM DiSC3(5) was then added. Subsequently, 10 μl of 2 M KCl was added to the mixture at 30S intervals, and the fluorescence was recorded every 3 s using an RF6000 fluorescence spectrophotometer (Shimadzu, Japan) with excitation and emission wavelengths of 645 and 665 nm, respectively. Each experiment was repeated three times.

### 2.9 Protease activity analysis

As described in the section 2.3, *P. americana* specimens were fed purified PBS or 0.125 mg·ml^-1^ or 0.031 mg·ml^-1^ Vip3Aa1 protein solution for 24 h.

Tryptase activity was assayed using N-a-benzoyl-DL-arginine-p-nitroanilide as substrate [14], glutathione S-transferase (GSH) activity was assayed using 2,4-Dinitrochlorbenzene (CDNB) as substrate [15], chymotrypsin-like proteinase activity was assayed using N-succinyl-alanine-alanineproline-phenylalanine p-nitroanilide (SAAPFpNA) as substrate [16], and α-naphyle esterase activity was assayed using α-NA as substrate [17]. For the assessment of all protease activities, the absorbance was read at 410 nm using an Elx808 microplate reader (BioTek, USA).

**Ethical approval**: The conducted research is not related to either human or animals use.

## 3 Results

### 3.1 Expression and purification of Vip3Aa1 in *E. coli* BL21(DE3)

A 2.37-kb fragment that was amplified by PCR from the *Bt* strain total DNA was cloned into the pMD-20 vector and completely sequenced, and a BLAST search identified the gene as *vip3Aa1* (GenBank accession number: JQ228435.1).

**Figure 1:**
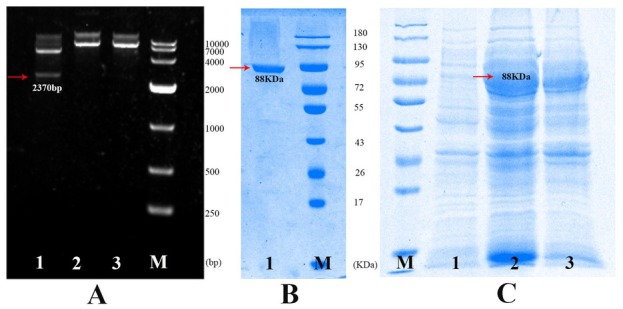
Expression and purification of Vip3Aa1 in *E. coli* BL21(DE3). Lane M: DNA marker; Lane 3: pET21b/BL21 digested with *BamH* I; Lane 3: pET21b/BL21 digested with *Xho* I; Lane 1: pET21b/BL21 digested with *BamH* I and *Xho* I. (B) Lane 1: purified Vip3Aa1 protein; Lane M: protein marker. (C) Lane M: protein marker; Lane 1: pET21b/BL21-induced whole-cell extract; Lane 2: pET21b-vip3Aa1/BL21-induced whole-cell extract; Lane 3: crude cytosolic extract.

After induction by IPTG, a novel protein with a size of approximately 88-kDa was found on the SDS-PAGE gel. After sonication of IPTG-induced *E. coli* (pET-21b/ Vip3Aa1), the supernatant proteins and precipitate were separated by centrifugation, and the 88-kDa protein was found to be mainly concentrated in the supernatant proteins **(Figure 1C**, Lane 2**).** In addition, Vip3Aa1 protein was soluble, and 88-kDa Vip3Aa1 was further purified by HisTrap resin **(Figure 1B)**.

### 3.2 Biological activity of Vip3Aa1 protein against *P. americana* and *B. germanica*

**Figure 2:**
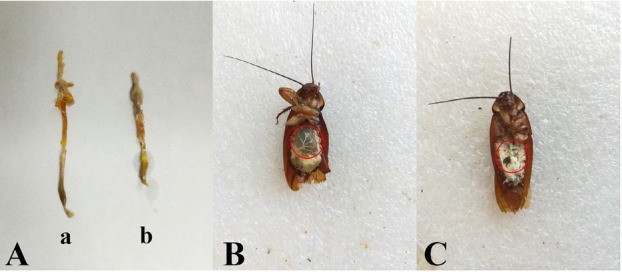
Insecticidal activity of Vip3Aa1 protein against *Periplaneta americana*. (A) Effect of Vip3Aa1 on the length of the alimentary system of *Periplaneta americana*: (a) *Periplaneta americana* fed PBS and (b) *Periplaneta americana* fed Vip3Aa1. (B) Image of *Periplaneta americana* after feeding on PBS Vip3Aa1.(C) Image of *Periplaneta americana* after feeding on PBS.

After feeding on Vip3Aa1 proteins, *P. americana* showed typical symptoms of poisoning, including reduced eating, vomiting, shaking, lack of balance while walking, overall paralysis, and death. An analysis of the alimentary system of *P. americana* revealed that the length of the alimentary system of the insects in the experimental group was significantly shortened compared with that of the control insects **(Figure 2A)**. Under normal circumstances, the cockroach crop is empty or full of food, but the crop of most of the cockroaches in the experimental group was obviously expanded and full of gas. No similar phenomenon has been reported previously **(Figure 2B and Figure 2C)**.

The Vip3Aa1 toxins were tested against adult *P. americana* and *B. germanica*. The estimated 12-h, 24-h, and 36-h LC_50_ values of Vip3Aa1 toward *P. americana* were approximately 0.703, 0.182, and 0.159 mg·ml^-1^, respectively, and the corresponding values against *B. germanica* were approximately 0.546, 0.276, and 0.118 mg·ml^-1^, respectively **(Table 1 and Table 2)**.

**Table 1:** Probit analysis for Vip3Aa1 toxins and Boric acid again *P. americana*

		Slope±SE	LC_50_	Range of 95% CL	Relative potency
12h	Boric acid	7.630±3.682	0.290g	0.233~1.852	1.0
	Vip3Aa1	1.567±0.325	0.703mg	0.425~1.372	412.0
24h	Boric acid	3.909±1.417	0.127g	0.059~0.175	1.0
	Vip3Aa1	2.147±0.386	0.182mg	0.119~0.281	697.0
36h	Boric acid	2.584±1.811	0.050g	2.709~0.104	1.0
	Vip3Aa1	2.255±0.414	0.159mg	0.105~0.243	314.0

**Table 2:** Probit analysis for Vip3Aa1 toxins and Boric acid again *B. germanica*

		Slope±SE	LC_50_	Range of 95% CL	Relative potency
12h	Boric acid	1.814±0.789	0.916g	0.364~5.394	1.0
	Vip3Aa1	1.480±0.300	0.546mg	0.324~1.025	1677.0
24h	Boric acid	1.602±0.615	0.481g	0.245~36.173	1.0
	Vip3Aa1	1.532±0.203	0.443mg	0.320~0.633	1085.0
36h	Boric acid	1.570±0.755	0.310g	0.180~5.701	1.0
	Vip3Aa1	1.815±0.351	0.118mg	0.060~0.215	2627.0

The biological activities of the Vip3Aa1 toxins were compared with that of boric acid, the Vip3Aa1 toxins were approximately 412.0, 697.0, 314.0-fold more potent than boric acid toward *P. americana* at 12-h, 24-h, and 36-h, respectively. At the same time points, they were 1677.0, 1085.0, 2627.0 -fold more potent toward *B. germanica*, respectively.

### 3.3 Vip3Aa1 proteolytic activation by *P. americana* gut-soluble proteases

**Figure 3:**
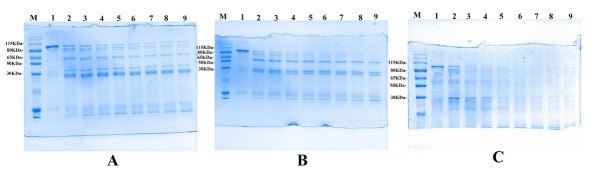
Proteolytic processing of Vip3Aa1 protein by trypsin, gut-soluble proteases and chymotrypsin. (A) Proteolytic processing of Vip3Aa1(1 mg·ml^-1^) protein by gut-soluble proteases (0.5 mg·ml^-1^) over times. (B) Proteolytic processing of Vip3Aa1(1 mg·ml^-1^) protein by chymotrypsin (0.250%) over time. (C) Proteolytic processing of Vip3Aa1(1 mg·ml^-1^) protein by trypsin (0.250%) over time. Lane M: M, protein marker; Lanes 1-7, incubation times of 0 min, 60 min, 120 min, 180 min, 240 min, 300 min, and 360 min, 420min, 480min respectively.

In vitro proteolysis studies were conducted to characterize both the stability and the processing of Vip3Aa1 protein in the midgut environment of *P. americana*. The 88-kDa Vip3Aa1 protein could be hydrolyzed into a 62-kDa protein core fragment by gut-soluble proteases **(Figure 3A)**, trypsin **(Figure 3C)** or chymotrypsin **(Figure 3B)**, and the three proteases showed similar kinetic activation patterns. In addition, the 62-kDa fragment was gradually degraded over time by the gut-soluble proteases. Vip3Aa1 protein exhibited significantly higher sensitivity to trypsin than to gut-soluble proteases and chymotrypsin.

### 3.4 P. americana intestinal HE and SEM

**Figure 4:**
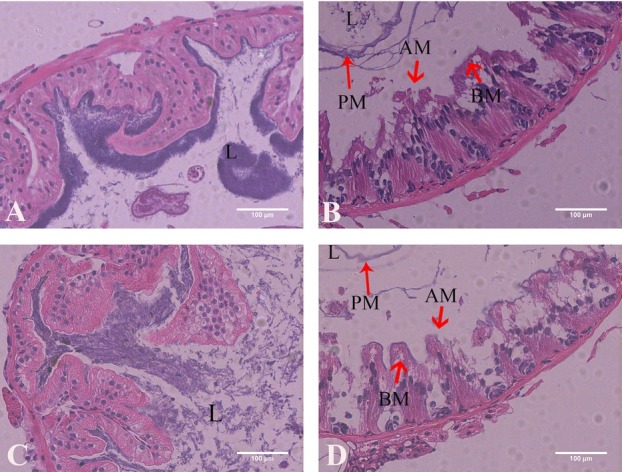
Histopathology (HE staining) of Vip3Aa1 in the midgut and columnar colon. (A) HE staining of the midgut of *Periplaneta americana* fed PBS (400×). (B) HE staining of the midgut of *Periplaneta americana* fed Vip3Aa1 (400×). (C) HE staining of the columnar colon of *Periplaneta americana* fed PBS (400×). (D) HE staining of the columnar colon of *Periplaneta americana* fed Vip3Aa1 (400×).

**Figure 5:**
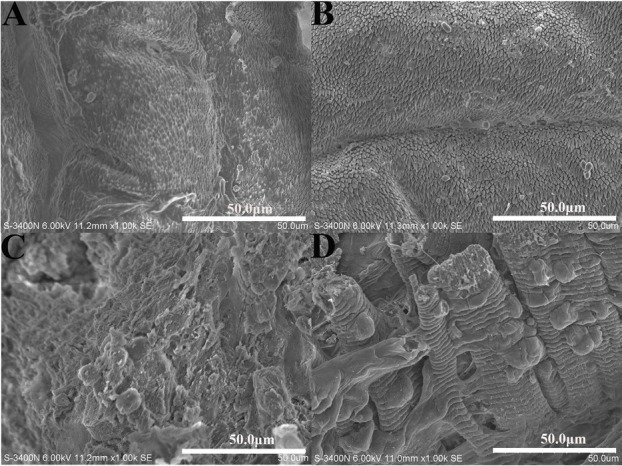
Histopathology (SEM) of Vip3Aa1 in the midgut and columnar colon. (A) SEM of the midgut of *Periplaneta americana* fed PBS (1000×). (B) SEM of the columnar colon of *Periplaneta americana* fed PBS (1000×). (C) SEM of the midgut of *Periplaneta americana* fed Vip3Aa1 (1000×). (D) SEM of the columnar colon of *Periplaneta americana* fed Vip3Aa1 (1000×).

The histopathological changes induced by Vip3Aa1 were observed by HE staining and SEM, and the results showed that the midgut and columnar colon epithelial cells were severely disrupted. Specifically, the peripheral membrane was wrinkled and shed from the surface of the epithelial cells, leading to the eventual rupture of individual or small groups of epithelial cells **(Figure 4)**. The SEM results showed that the epithelium was severely damaged, the thorns on the epithelium surface had disappeared, and the epithelium was shed. These histopathological changes contributed to the death of *P. americana* **(Figure 5)**.

### 3.5 Effect of the Vip3Aa1 protein on P. americana proteolytic activity

We tested four types of enzymes in the midgut, including two digestive enzymes (trypsin and chymotrypsin) and two detoxifying enzymes (glutathione S-transferase and α-NA esterase) **(Table 3, 4, 5** and **6)**. As indicated by one way ANOVA, the activities of trypsin and chymotrypsin, glutathione S-transferase, α-NA esterase were significantly different among 0.125 mg·ml^-1^ Vip3Aa1 group, 0.031 mg ml^-1^ Vip3Aa1 group and the control group. The activities of trypsin and chymotrypsin were significantly lower in the control group than in the groups treated with 0.125 mg·ml^-1^ and 0.031 mg ml^-1^ Vip3Aa1 (p<0.05). In addition, the activities of glutathione S-transferase and α-NA esterase in the control group were significantly lower than those in the groups treated with 0.125 mg·ml^-1^ and 0.031 mg ml^-1^ Vip3Aa1 (p<0.05).

**Table 3:** Effect of Vip3Aa1 protein on tryptase

Groups	n	$\bar{x}\pm s$	F	P
Control group	10	0.462±0.0789		
0.125 mg·ml^-1^ group	10	0.818±0.0604	107.1	p＜0.01
0.031 mg·ml^-1^ group	10	0.820±0.045		

**Table 4:** Effect of Vip3Aa1 protein on chymotrypsin-like proteinase in *Periplaneta americana*

Groups	n	$\bar{x}\pm s$	F	P
Control group	10	0.418±0.097		
0.125 mg·ml^-1^ group	10	1.203±0.262	64.33	p＜0.01
0.031 mg·ml^-1^ group	10	1.351±0.198		

**Table 5:** Effect of Vip3Aa1 protein on glutathione S-transferase

Groups	n	$\bar{x}\pm s$	F	P
Control group	10	0.123±0.018		
0.125 mg·ml^-1^ group	10	0.215±0.013	123.8	p＜0.01
0.031 mg·ml^-1^ group	10	0.234±0.019		

**Table 6:** Effect of Vip3Aa1 protein on α-NA esterase

Groups	n	$\bar{x}\pm s$	F	P
Control group	10	0.068±0.008		
0.125 mg·ml^-1^ group	10	0.116±0.006	168.6	p＜0.05
0.031 mg·ml^-1^ group	10	0.120±0.007		

### 3.6 Immunolocalization analysis

**Figure 6:**
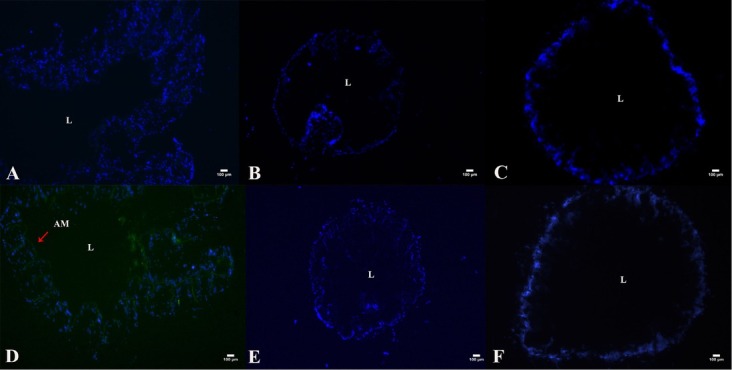
Immunolocalization of J-Vip3Aa1 in crop, midgut, and columnar colon tissue sections. (A) Crop (400× magnification). (B) Midgut (400× magnification). (C) Columnar colon (400× magnification). (D) Crop incubation with J-Vip3Aa1(400× magnification). (E) Midgut incubation with J-Vip3Aa1(400× magnification). (F) Columnar colon incubation with J-Vip3Aa1(400× magnification). AM, apical membrane; L, gut lumen.

The present study showed that Vip3Aa1 protein can bind to certain receptors in the midgut to exert toxic effects [18]. To test whether J-Vip3Aa1 binds to the columnar colon, midgut or crop, J-Vip3Aa1 was labeled with the fluorescent dye fluorescein isothiocyanate (FITC), and purified FITC-J-Vip3Aa1 protein was confirmed using a fluorescence imaging system. Sections from *P. americana* were observed under a fluorescence microscope, and an immunolocalization analysis revealed no green fluorescence either inside the cells or in the apical membrane of the columnar colons and midgut **(Figure 6E & Figure 6F)**. Surprisingly, green fluorescence was observed along the entire crop apical surface, which indicated that J-Vip3Aa1 can bind to a receptor in the crop **(Figure 6D)**.

### 3.7 Pore formation activity of Vip3Aa1

**Figure 7:**
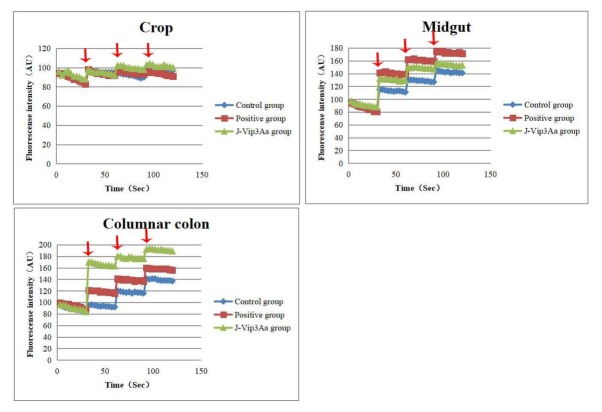
Formation of an ion channel on BBMVs from *Periplaneta americana* by J-Vip3Aa1. (A) Crop. (B) Midgut. (C) Columnar colon. BBMVs were incubated with buffer, 100 μM valinomycin or 4 μg/ml J-Vip3Aa at 4°C for 30 min, and 6 μl of 1 mM DiS-C3 was then added (5). KCl was added at the times indicated with arrows. Each experiment was repeated three times.

As shown in **Figure 7**, the fluorescence intensity in the crop exhibited no changes. Because BBMVs exhibit an intrinsic permeability to K^+^, KCl was added to the midgut or columnar colon BBMV mixture at different time points, and an increase in fluorescence intensity was detected in the buffer-treated BBMVs. Valinomycin, as a K^+^ ionophore, significantly enhanced the permeability to K^+^ of BBMVs in the midgut and columnar colon. The increase in permeability was notably greater in the presence than in the absence of J-Vip3Aa1, slightly smaller than that obtained with valinomycin in the midgut and greater than that obtained with valinomycin in the columnar colon.

## 4 Discussion

Vips, a new type of broad-spectrum insecticidal protein whose mechanisms differ from those of Cry1A toxins, might become a new approach for controlling pests. Vips are produced during the vegetative growth phase of *Bt* and are highly stable. To date, 15 variants of Vip1 proteins, 20 variants of Vip2 proteins and 101 variants of Vip3 proteins have been identified [19]. In this study, a *vip3Aa1* gene was amplified from a *Bt* strain preserved in our laboratory. Based on its sequence and the results of subsequent BLAST searches, the gene was identified as *vip3Aa1* (GenBank accession number: JQ228435.1). Vip3Aa1 protein is toxic to many *Lepidoptera* species, such as *Ephestia kuehniella*, *Prays oleae, Spodoptera frugiperda* [20], *Helicoverpa armigera* [21], *Spodoptera litura* [22], and *Spodoptera exigua* [23], but we did not find any reports describing the activity of Vip3Aa1 against *P. americana* or *B. germanica*.

Damage to the *P. americana* midgut and columnar colon epithelium was clearly induced by Vip3Aa1. Previous studies have not observed any damage in the crop, columnar colon, or midgut of non-susceptible insects, but in our study, both HE staining and SEM revealed that Vip3Aa1 caused severe damage to the columnar colon and midgut. Midgut is considered as the most important site for terminal digestion and absorption of nutrition.

The proventriculus of *P. americana*, which connects the midgut and crop, bears on its inner surface a series of six large, radially arranged chitinous teeth surrounded by a heavy ring of circular muscles. When the circular muscles contract, the six teeth entirely occlude the lumen between the midgut and the crop [24]. We thus speculate that Vip3Aa1 might induce circular muscle contractions, and as result, food and water cannot enter the midgut, resulting in crop expansion. However, the mechanism underlying this phenomenon is unclear.

The mechanism underlying the insecticidal action of Vip3Aa1 protein is unclear. Both Vip3Aa1 and crystal proteins have a similar mode of insecticidal action: (1) the full-length toxin is hydrolyzed into active toxin by midgut proteases; (2) the toxin binds to a specific receptor in midgut cells; (3) pore formation occurs; (4) and the midgut cells burst, causing insect death.

Based on previous studies, Vip3Aa1 proteins need to be hydrolyzed into active proteins by gut-soluble proteases, and unactivated proteins cannot form pores in vitro. However, full-length Vip3Aa1 can be hydrolyzed by both susceptible and non-susceptible insects [25]. In addition, that Vip3Aa1 can be activated into 55-70-kDa toxic peptides by a variety of alkaline proteases, such as trypsin, chymotrypsin, elastase, and thiol protease. Thus, the proteolytic step is not a determining factor for insect specificity but rather serves as an activation step. In our study, the 88-kDa Vip3Aa1 protein was incubated with trypsin, chymotrypsin, or *P. americana* gut-soluble proteases for different incubation times, as shown in **Figure 3**. A dominant stable 62-kDa protein is obtained by the action of gut-soluble proteases, trypsin, or chymotrypsin, which is consistent with the published literature. Vip3Aa1 was more sensitive to proteolytic activation by trypsin than to that by chymotrypsin or gutsoluble proteases. The 62-kDa protein was unstable and broke down even before all the pro-toxin was processed [26].

The pore formation activity directly reflects the toxicity of J-Vip3Aa1. To assess the ability of J-Vip3Aa1 to form ion channels on BBMVs from *P. americana*, the permeability of BBMVs to K^+^ was examined using the voltage-sensitive cyanine dye DiSC3(5), and we discovered that J-Vip3Aa1 is able to form pores in the midgut and columnar colon but not in the crop. The pore formation activity observed in vitro can likely account for the documented histological changes in the midgut, columnar colon and crop. Because the three-dimensional structure of J-Vip3Aa1 has not yet been resolved, the mechanism through which Vip3Aa1 promotes pore formation remains unclear. Primary sequence divergence and an examination of the predicted secondary structure have indicated that the channels of Vip3Aa1 differ from those of Cry proteins [27]. In future research, the three-dimensional structure of Vip3Aa1 protein will be studied to hopefully clarify its mechanism of insecticidal action.

Most studies have not considered toxin activation to be a determining factor for insect specificity, and the toxicity of Vip3Aa1 protein depends on its ability to bind to specific receptors in the midgut. It remains unknown whether activated Vip3Aa1 protein has an appropriate receptor in the *P. americana* digestive tract. To test whether J-Vip3Aa1 can bind to the *P. americana* digestive tract, J-Vip3Aa1 was labeled with FITC. As shown in Figure 7, in contrast to previous studies [28], FITC-J-Vip3Aa1 did not bind to the midgut or columnar colon but bound to the crop, but no damage was observed in the crop. Unlike for Cry proteins, no Vip3Aa1-binding protein has been identified to date, and previous studies have not provided any direct evidence showing that Vip3Aa1 exerts insecticidal activity by binding to receptors. Mi Kyong Lee hypothesized that the pore-forming properties of Vip3Aa1 alone might account for its observed toxicity and did not preclude the possibility that unique binding events might mediate other aspects of Vip3A bioactivity [27]. In Kun Jiang’s study, Vip3Aa1 induced the apoptosis of Sf9 cells through mitochondrial-mediated and caspase-dependent pathways [29]. The study performed by Patricia Hernández Martínez revealed that the exposure of *S. exigua* larvae to sublethal concentrations of Vip3Ca triggered caspase-dependent pathways and led to apoptotic cell death [30]. We hypothesize that activated Vip3Aa1 does not need to bind to a receptor and can directly damage the midgut and columnar colon.

Vip3Aa1 is mainly activated by trypsin and chymotrypsin, the midgut protease of insects is closely related to the production of resistance. If the hydrolysis activity of trypsin and chymotrypsin is significantly reduced or significantly increased by gene mutation, the Vip3Aa1 will deficient proteolysis or over-hydrolyzed, this will lead to increased resistance of insects to Vip3Aa1[31, 32]. The activities of trypsin and chymotrypsin in the Vip3Aa1-treated groups were significantly increased, and this result was consistent with that reported by Feifei Song [22], who analyzed the transcriptional profile of *Spodoptera litura* larvae fed Vip3Aa1 and found that most trypsin and chymotrypsin genes were upregulated. Other studies have revealed that increased trypsin and chymotrypsin activities could reduce insect resistance to Cry protein [17, 33].

Glutathione S-transferase is known to be involved in the metabolization of various endogenous compounds, but is also recognized as one of the major mechanisms conferring insecticide resistance in many pests. Biochemical data revealed an induction in Glutathione S-transferase activities confirming the observation previously reported in *Spodoptera frugiperda* [34].

Glutathione S-transferase and α-NA esterase help protect cockroaches against toxic and foreign substances, but the approaches used by cockroaches to detoxify toxic proteins have not been investigated. In the present study, we found that the activities of glutathione S-transferase and α-NA esterase in the *P. americana* midgut were increased after the cockroaches fed on Vip3Aa1. Significantly increased midgut α-NA esterase activity has been associated with *Bt* resistance in some lepidopteran pests [35], and α-NA esterase has the ability to bind to and detoxify Cry1Ac. The detoxification protein glutathione S-transferase was strongly upregulated in *Spodoptera exigua* after the insect fed on Vip3Aa1 [22].

In conclusion, we demonstrated the novel finding that Vip3Aa1 protein exhibits activity against *P. americana* and *B. germanica*. In addition, we found that the mechanism through which Vip3Aa1 kills cockroaches might differ from that through used by the protein to kill lepidopteran insects. Vip3Aa1 protein does not need to bind to receptors and can directly exert its toxic effects, and the Vip3Aa1 toxin was thus found to be effective for the control of cockroaches. These results highlight the usefulness of Vip3Aa1 for cockroach control.
